# Stroke, Thrombosis, Bleeding and Addiction to Anticoagulants in the Context of Course Therapy: A Pharmacologic Perspective

**DOI:** 10.31083/j.rcm2307236

**Published:** 2022-06-24

**Authors:** Aleksandr Urakov, Anastasia Stolyarenko, Ilnur Yagudin, Nikita Mukhutdinov, Ilnur Bashirov

**Affiliations:** ^1^Department of General and Clinical Pharmacology, Izhevsk State Medical Academy, 426034 Izhevsk, Udmurt Republic, Russia; ^2^Department of Modeling and Synthesis of Technological Structures, Institute of Mechanics, Udmurt Federal Research Center, 426067 Izhevsk, Udmurt Republic, Russia

**Keywords:** drug, heart, blood, stroke, bleeding, patient

## Abstract

After reading with great interest the article entitled: “Non-vitamin K antagonist oral anticoagulants (NOACs) do not increase the risk of hepatic impairment in patients with non-valvular atrial fibrillation: insights from multi-source medical data” authored by Zhi-Chun Gu *et al*. and published by Reviews in Cardiovascular Medicine, we would like to add the following thoughts. Oral anticoagulants are generally accepted in patients with non-valvular atrial fibrillation to prevent thrombosis and stroke. Since anticoagulants are taken daily for many months in these patients, we cannot rule out chronic poisoning and the development of liver failure. But another complication is just as likely, that being bleeding. Thus, the determining risk factor for the health of patients with a prolonged course of oral anticoagulants is hypofunctional activity of the blood coagulation system, which remains at the same level throughout the course of treatment. At the same time, it is the activity of the blood coagulation system that is an important and very sensitive link of adaptation to various external and internal factors, including anticoagulants. The fact is that regular and prolonged oral use of anticoagulants is likely to develop and tolerance to them. That is why it is necessary to carefully study the relationship between the dose of oral anticoagulants, the duration of pharmacotherapy and the development of thrombosis (bleeding) in patients with non-valvular atrial fibrillation.

Zhi-Chun Gu *et al*. [1] conducted an interesting study of non-vitamin K antagonist oral anticoagulants (NOACs) in their article published in the journal Reviews in Cardiovascular Medicine but we would like to add the following thoughts. Long-term oral anticoagulants are recommended for thrombosis and stroke prevention, which not only reduces the likelihood of thrombosis, but also increases the likelihood of bleeding [2, 3]. Therefore, it is the activity of the patient’s blood plasma coagulation system that determines the effectiveness and safety of long-term oral use of anticoagulants. At the same time, the effectiveness and safety of NOACs is determined by the dose administered and by the the patient’s body weight. The following pattern is characteristic: an increasing daily and course dose of NOACs improves protection against thrombosis while simultaneously increasing the risk of bleeding. In patients with a body weight greater than 70 kg, the effectiveness of thrombosis prevention and the likelihood of bleeding with pharmacotherapy with equal daily and course doses of NOACs is reduced compared to patients with a lower body weight [4, 5]. Consequently, analysis of the efficacy and safety of pharmacotherapy with oral anticoagulants without taking into account their dose, duration of treatment and body weight of patients is not accurate.

It is generally accepted that lower doses of NOACs and shorter courses of treatment are less dangerous when compared to higher doses of NOACs and longer courses of treatment [5]. First, nonvalvular atrial fibrillation can be accompanied by heart failure, which can alter blood flow, liver function and hepatic metabolism of NOACs. In this case, the likelihood of heart and liver failure as well as changes in clotting system activity results in an increased risk of treatment with NOACs. Consequently, these complications may not be present with a short course of treatment with oral anticoagulants.

Second, change in liver function can alter the synthesis of natural anticoagulants in the patient, which can affect the effectiveness and safety of NOACs initially administered in the ‘correct’ doses [6]. In particular, an increase in heart and liver failure, often observed with increasing disease duration, may reduce the effectiveness of thrombosis prevention by pharmacotherapy with NOACs. In this case, hepatic insufficiency may be an aggravating factor in the relative ineffectiveness of NOACs.

Third, increasing the duration of regular NOACs administration can result in tolerance to the drug [7, 8]. Therefore, the efficacy and safety of NOACs may be altered if the duration of treatment with oral anticoagulants is increased. It is likely that the efficacy of NOACs will decrease with increasing duration of treatment if doses are not increased. This results in clinicians often having to increase single and daily doses of NOACs as the duration of treatment increases [9]. In turn, increasing doses can increase their toxic effects on the liver.

It is an accepted phenomena that increasing the duration of daily intake of drugs can result in tolerance, thereby reducing the effectiveness of medication over time and potentially eliminating any pharmacologic effectiveness of the drug. Moreover, further regular intake of medications potentially makes patients dependent on them [5, 10].

Finally, it should be noted that there is currently no standard for evaluating the efficacy and safety of pharmacotherapy with oral anticoagulants [11]. This situation explains why researchers use different schemes to evaluate the effectiveness of NOACs. In addition, the lack of a unified standard for evaluating the clinical efficacy and safety of NOACs explains why researchers have difficulty clarifying the relationship between the efficacy and safety of NOACs with the dose administered, duration of treatment and patient’s body weight [12].

We suggest the need to develop a standard for evaluating the efficacy and safety of NOACs for the prevention of thrombosis and stroke, so that all investigators apply a unified study design which includes a range of doses administered along with duration of treatment based on the patient’s body weight and age.

## References

[1] Gu ZC, Wang J, Zhang C, Zhao B, Li ZL (2022). Non-vitamin K antagonist oral anticoagulants (NOACs) do not increase the risk of hepatic impairment in patients with non-valvular atrial fibrillation: insights from multi-source medical data. *Reviews in Cardiovascular Medicine*.

[2] Wu J, Zhang Y, Liao X, Lei Y (2020). Anticoagulation therapy for non-valvular atrial fibrillation: a mini-review. *Frontiers in Medicine*.

[3] Kaide CG, Gulseth MP (2020). Current Strategies for the Management of Bleeding Associated with Direct Oral Anticoagulants and a Review of Investigational Reversal Agents. *The Journal of Emergency Medicine*.

[4] Lee S, Choi E, Park CS, Han K, Jung J, Oh S (2019). Direct Oral Anticoagulants in Patients with Nonvalvular Atrial Fibrillation and Low Body Weight. *Journal of the American College of Cardiology*.

[5] Ritter JM, Flower R, Henderson G, Loke YK, MacEwan D, Rang HP (2020). *Rang and Dale’s Pharmacology. 9th edn.*.

[6] Buliarca A, Horhat A, Mocan T, Craciun R, Procopet B, Sparchez Z (2021). Viscoelastic tests in liver disease: where do we stand now. *World Journal of Gastroenterology*.

[7] Boutitie F, Pinede L, Schulman S, Agnelli G, Raskob G, Julian J (2011). Influence of preceding length of anticoagulant treatment and initial presentation of venous thromboembolism on risk of recurrence after stopping treatment: analysis of individual participants’ data from seven trials. *British Medical Journal*.

[8] Lobastov KV (2019). Contemporary approaches to determine the duration of anticoagulant therapy for venous thromboembolism. *Pirogov Russian Journal of Surgery*.

[9] Kimura S, Toyoda K, Yoshimura S, Minematsu K, Yasaka M, Paciaroni M (2022). Practical “1-2-3-4-Day” rule for starting direct oral anticoagulants after ischemic stroke with atrial fibrillation: combined hospital-based cohort study. *Stroke*.

[10] Brenner GM, Stevens CW (2018). *Pharmacology. 5th edn.*.

[11] Steffel J, Collins R, Antz M, Cornu P, Desteghe L, Haeusler KG (2021). 2021 European heart rhythm association practical guide on the use of non-vitamin K antagonist oral anticoagulants in patients with atrial fibrillation. *Europace*.

[12] Daly AK, King BP (2003). Pharmacogenetics of oral anticoagulants. *Pharmacogenetics*.

